# Combining proximal and remote sensing to assess ‘Calatina’ olive water status

**DOI:** 10.3389/fpls.2024.1448656

**Published:** 2024-08-20

**Authors:** Alessandro Carella, Roberto Massenti, Francesco Paolo Marra, Pietro Catania, Eliseo Roma, Riccardo Lo Bianco

**Affiliations:** Department of Agricultural, Food and Forest Sciences (SAAF), University of Palermo, Palermo, Italy

**Keywords:** precision irrigation, drought stress, *Olea europaea* L., crop water stress index, normalized difference vegetation index, stem water potential, UAVs

## Abstract

Developing an efficient and sustainable precision irrigation strategy is crucial in contemporary agriculture. This study aimed to combine proximal and remote sensing techniques to show the benefits of using both monitoring methods, simultaneously assessing the water status and response of ‘Calatina’ olive under two distinct irrigation levels: full irrigation (FI), and drought stress (DS, -3 to -4 MPa). Stem water potential (Ψ_stem_) and stomatal conductance (g_s_) were monitored weekly as reference indicators of plant water status. Crop water stress index (CWSI) and stomatal conductance index (Ig) were calculated through ground-based infrared thermography. Fruit gauges were used to monitor continuously fruit growth and data were converted in fruit daily weight fluctuations (ΔW) and relative growth rate (RGR). Normalized difference vegetation index (NDVI), normalized difference RedEdge index (NDRE), green normalized difference vegetation index (GNDVI), chlorophyll vegetation index (CVI), modified soil-adjusted vegetation index (MSAVI), water index (WI), normalized difference greenness index (NDGI) and green index (GI) were calculated from data collected by UAV-mounted multispectral camera. Data obtained from proximal sensing were correlated with both Ψ_stem_ and g_s_, while remote sensing data were correlated only with Ψ_stem_. Regression analysis showed that both CWSI and Ig proved to be reliable indicators of Ψ_stem_ and g_s_. Of the two fruit growth parameters, ΔW exhibited a stronger relationship, primarily with Ψ_stem_. Finally, NDVI, GNDVI, WI and NDRE emerged as the vegetation indices that correlated most strongly with Ψ_stem_, achieving high R^2^ values. Combining proximal and remote sensing indices suggested two valid approaches: a more simplified one involving the use of CWSI and either NDVI or WI, and a more comprehensive one involving CWSI and ΔW as proximal indices, along with WI as a multispectral index. Further studies on combining proximal and remote sensing data will be necessary in order to find strategic combinations of sensors and establish intervention thresholds.

## 1 Introduction

In recent years, climate change has created serious problems regarding the availability and use of water in agriculture, especially in areas characterized by scarce rainfall and high temperatures during the summer (Konapala et al., 2020; Pokhrel et al., 2021). These conditions of severe drought are occurring in the Mediterranean basin, creating significant challenges for local agriculture (Carella et al., 2023).

Deficit irrigation strategies have been demonstrated to be effective methods for achieving efficient water use in regions facing water scarcity (Nikolaou et al., 2020). The objective of implementing a deficit irrigation strategy is to achieve satisfactory crop yields by supplying the crop with a reduced irrigation volume compared to its potential water requirements (Tong et al., 2022). Growing crops that are well-adapted to water deficit conditions (deficit irrigation) is one of the strategies to achieve satisfactory productivity without the overuse of water resources (Gómez-Bellot et al., 2024). In this context, the olive tree (*Olea europaea* L.) emerges as a highly resilient species to water stress, with its production being influenced not only by climatic factors but also by management practices (Massenti et al., 2022b).

Another important aspect of current olive cultivation is the trend toward adopting high-density (HD) planting systems and mechanizing cultural operations. Combining the advantages of comprehensive mechanization, precision agriculture techniques, and the use of local cultivars that well adapt to the climate and soil can greatly reduce production costs associated with labor expenses (Lo Bianco et al., 2021). An olive cultivar that fits well modern growing systems (i.e., high planting density and level of mechanization) is ‘Calatina’, a minor Sicilian cultivar recently rediscovered thanks to its low vigor and high yield efficiency. ‘Calatina’ also possesses a degree of shoot bending and branching density suitable for HD and super-high-density (SHD) planting systems along with high harvesting efficiency (large fruits), demonstrating similar or better productive performance than ‘Arbequina’, the most widespread cultivar for HD and SHD systems (Caruso et al., 2021; Carella et al., 2022; Massenti et al., 2022a). Under HD and SHD systems, water needs and irrigation management become crucial to reach optimum yields and high quality final products. However, there are no studies on ‘Calatina’ water status assessment and irrigation management.

Studying how olive orchards respond to external influences presents significant challenges due to the diverse range of environments across the Mediterranean and the wide variety of planting systems employed. Prolonged periods of drought stress can trigger various physiological responses in olive trees. These include the closure of stomata (Mairech et al., 2021; Carella et al., 2023), limitation of photosynthesis (Melaouhi et al., 2021), decreased gas exchange (Pierantozzi et al., 2020), and osmotic adjustments (Scalisi et al., 2020; Abboud et al., 2021; Marino et al., 2021). When favorable water conditions return after a period of water deficit, olive trees undergo partial recovery in processes such as transpiration, photosynthesis, chlorophyll fluorescence indices, and osmotic potential (Connor, 2005; Fernandez, 2014). This implies that while tissue water content may rapidly increase, leaf function may take several days to fully restore, depending on the severity of the stress and cultivar-specific restoring capacity (Fernandez, 2014; Scalisi et al., 2020; Wahab et al., 2022).

The timing of irrigation events can be supported by data related to either soil moisture content or plant water status (Bazzi et al., 2019). The established conventional method for assessing plant water status is by measuring Ψ_stem_ using Scholander’s pressure chamber. However, this method is labor-intensive and time-consuming, and requires high skills by operators. In recent years, technological advancements have enabled the testing and introduction of various plant-based sensors for assessing plant water status and improve irrigation water management in orchard systems. While many of these sensors do not directly measure plant water status, they monitor specific physiological processes that demonstrate varying correlations with plant water status (Carella et al., 2024). However, due to the multitude of factors influencing tree physiological responses, such as tree phenological stage, environmental conditions, and genotype-specific traits, the development of simplified and standardized water management protocols using these sensors remains complex (Cocozza et al., 2015; Scalisi et al., 2020). In the meantime, remote and proximal sensing technologies for assessing field variability are becoming increasingly common in precision agriculture. This trend is propelled by their relatively lower costs and non-invasive nature compared to conventional methods (Caruso et al., 2022a).

The use of proximal sensing techniques, such as infrared thermography (Ben-Gal et al., 2009) and daily fruit diameter variation by fruit gauges (Morandi et al., 2007; Boini et al., 2019; Giovannini et al., 2022; Khosravi et al., 2022; Carella et al., 2023), are potentially effective and continuous methods to assess plant water status. Scalisi et al. (2020) observed that coupling Ψ_stem_ with fruit gauges and leaf patch clamp pressure probes (LPCP probes) data can be a reliable tool for evaluating fruit tree water status and smarter irrigation management. Moreover, García-Tejero et al. (2018) determined that the infrared thermography approach has a great advantage due to the large amount of information provided. The use of infrared thermography has proved to be a suitable technique for monitoring the water status of fruit trees (Egea et al., 2016; García-Tejero et al., 2018; Blanco et al., 2023). In details, the most utilized method for plant water status assessment involves the normalization of canopy temperature through the calculation of some indices, e.g., crop water stress index (CWSI) and stomatal conductance index (Ig) (Jackson et al., 1981; Idso, 1982; Jones et al., 2002).

Remote sensing techniques enable the rapid detection of spatial variability of tree water status over large areas using thermal, multispectral and hyperspectral cameras (Carella et al., 2024). Among the most common platforms used in remote sensing for detailed scale irrigation management are Unmanned Aerial Vehicles (UAVs) (Roma and Catania, 2022). Several studies have found that remote sensing over olive orchards can provide an accurate estimation of tree water status. Most studies emphasize that remote sensing with thermal cameras is highly correlated with plant water status (Ben-Gal et al., 2009; Egea et al., 2016; Caruso et al., 2022b). However, these techniques have some limitations, mainly related to the calculation method used to determine the CWSI (Berni et al., 2009). Nonetheless, detection of spectral plant condition also allows for an estimation of plant water status (Marino et al., 2014; Rallo et al., 2014; Marques et al., 2023). Indeed, several studies have obtained correlations between various vegetation indices and plant water conditions. Among the most used indices, we find normalized difference vegetation index (NDVI, Rouse et al., 1974), normalized difference RedEdge (NDRE, Maccioni et al., 2001), and indices that utilize the Short Wave InfraRed (SWIR) band. Although the latter indices are very efficient, they cannot be obtained from normal multispectral cameras, but only from spectroradiometers or hyperspectral cameras (Herrmann et al., 2010). For this reason, water stress conditions are increasingly being investigated and detected with normal multispectral cameras, as they are cheaper and easier to use.

The combined use of proximal and remote sensing techniques can offer a more comprehensive and accurate indication of plant water status and irrigation requirements. Proximal sensors provide accurate and continuous real-time data for individual plants, while data from UAVs or satellites can extend coverage across the field (Matese et al., 2018; Jin et al., 2022; Carella et al., 2024). Remote sensing data provide valuable information on spatial variability through effective field mapping, allowing the strategic positioning of proximal sensors only in certain areas of the field (Alexopoulos et al., 2023; Roma et al., 2023). Data collected by soil electrical conductivity sensors and UAVs were used by Caruso et al. (2022) to delineate homogeneous zones within a densely irrigated olive orchard. They observed that tree water use efficiency (WUE) varies according to the placement within the orchard. Furthermore, they found that tree vigor emerges as a predominant factor affecting the final fruit yield under optimal water availability.

On these bases, this study aims to combine proximal and remote sensing techniques to show the benefits of using both monitoring methods, while simultaneously assessing the water status and response of ‘Calatina’ olive under two distinct irrigation regimes.

## 2 Materials and methods

**Figure 1:**
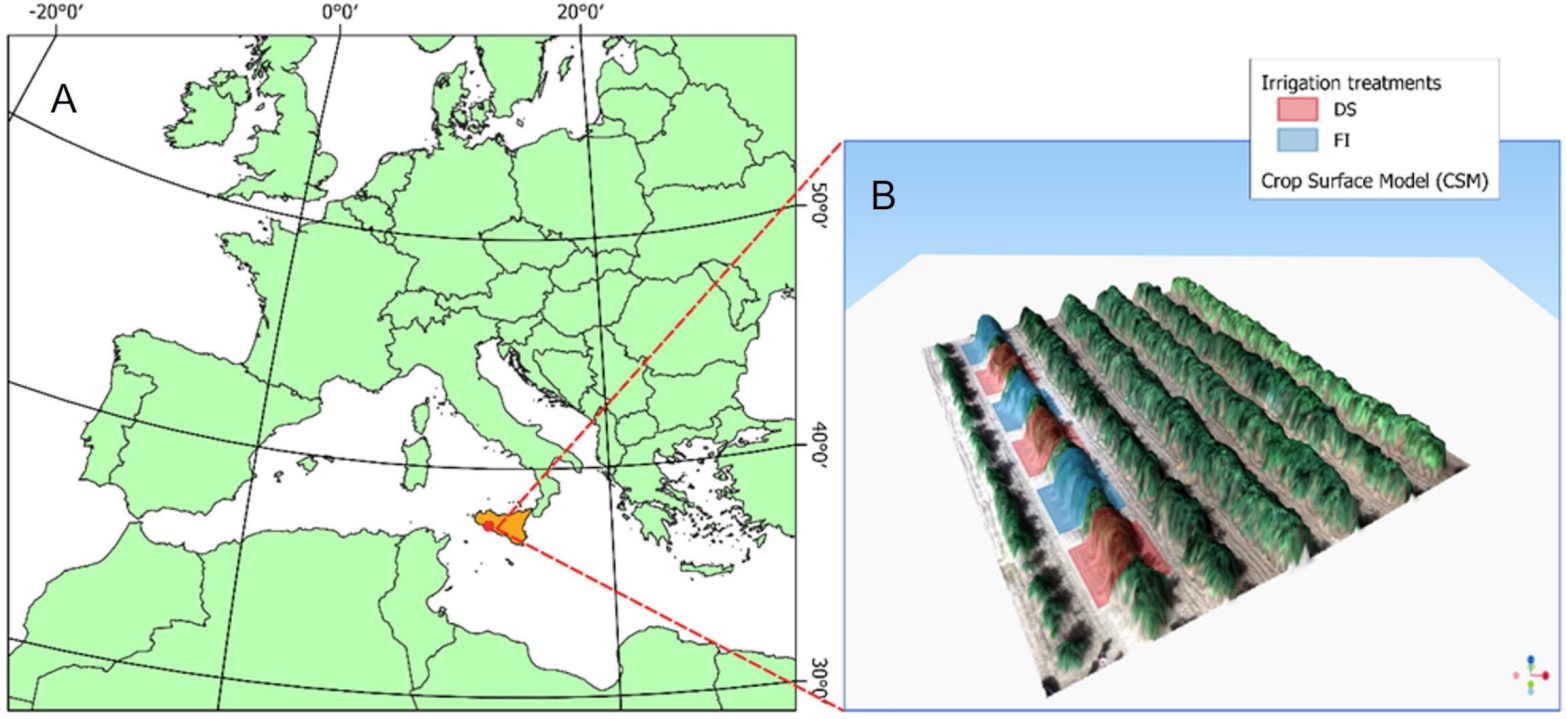
Experimental site location **(A)**; Experimental plot with Crop Surface Model (CSM) of the olive orchard, and full irrigation (FI) and drought stress (DS) treatments design **(B)**.

The experiment was conducted from summer to fall 2023, in a high-density olive orchard (6 x 2 spacing, 833 trees/ha) located near Sciacca (37°29’ N and 13°12’ E, 138 m a.s.l., **Figure 1A**). Eleven-year-old own-rooted trees were trained to “free palmette” along North-South-oriented hedgerows. The Sicilian cultivar Calatina was selected as potentially suitable for new high-density plantings mainly due to its low vigor, high productivity and good olive oil quality (Massenti et al., 2022a). The soil is a sandy-clay-loam (60% sand, 18% silt, 22% clay), with pH 7.7 and low active carbonates (<5%). Trees were regularly fertilized and pruned according to ordinary practices.

Air temperature (T) and relative humidity (RH) were measured at one-hour interval using an Elitech RC-51H data-logger (Elitech, London, UK), placed in the orchard within the experimental plot. RH (%) and T (°C) data were used to calculate vapor pressure deficit (VPD, kPa) with the following equation (Chazarreta et al., 2023):


$$VPD=VP_{s}-VP_{a}$$

where:


$$VP_{s}(saturatedvaporpressure)=0.6108^{\frac{17.27T}{T+237.3}}$$

$$VP_{a}(actualvaporpressure)=\frac{RH}{100VP_{s}}$$

Trees were irrigated weekly using two self-compensating in-line drippers per tree, each delivering 16 L/h. Two irrigation levels were imposed, selected based on two ranges of stem water potential: Full Irrigation (FI), maintaining the tree water potential at approximately -1.5 to -2.5 MPa (conventional farm management), and Drought Stress (DS), maintaining the tree water potential within the range of -3.5 to -4 MPa, according to the thresholds defined in Marra et al. (2016) and Marino et al. (2018). Nine plants per treatment were selected and divided into three blocks of three plants each (**Figure 1B**). A buffer plant separates each block to avoid influence between treatments.

Measurements were carried out at stages III (cell expansion) and IV (maturation) of fruit development.

### 2.1 Plant water status

Midday stem water potential (Ψ_stem_; MPa) was the main reference for evaluation of plant water status. Ψ_stem_ was measured with a Scholander’s pressure chamber (PMS 1000, Instrument Company, Albany, OR, USA). Measurements were taken at around 13:00 on four shoots per plant, each covered with aluminum foil 1 hour before measurement (Moriana et al., 2012). Leaf stomatal conductance (g_s_; mmol H_2_O m^-2^ s^-2^) and net photosynthesis (P_n_; µmol CO_2_ m^-2^ s^-2^) were measured weekly using a portable gas exchange system CIRAS-2 (PP Systems^®^, Hitchin, UK) on two sun-exposed leaves per plant (Marino et al., 2018). Both parameters were measured once a week, specifically on 14 July, 1, 8, 16, 22 and 29 August; 6, 11, 19 and 26 September; and 3 and 10 October.

### 2.2 Proximal sensing measurements

#### 2.2.1 Thermal imaging

Infrared thermal images were taken with a FLIR i7 hand-held thermal camera (FLIR systems, Wilsonville, Oregon, USA), with a resolution of 140 x 140 pixels and a spectral range of 7.5–13 μm. The camera has a thermal sensitivity of 0.1°C and an accuracy of ± 2%. Emissivity was set at 0.98, according to Rubio et al. (1997). Thermal images were taken weekly, at the same time of the other measurements. Each image included the canopy of each individual tree, along with a fully transpiring reference (T_wet_, as a non-water stressed baseline) and a non-transpiring reference (T_dry_). The references were obtained following the methodology proposed by Jones et al., 2002. T_wet_ was determined using leaves sprayed with water and a drop of detergent (0.01% v/v) (Fuentes et al., 2012) 1 minute before taking the thermal image as wet references. For T_dry_, leaves were covered with petroleum jelly about 1 h before measurement to artificially close stomata and inhibit transpiration. Crop water stress index (CWSI) was calculated using the following equation:


$$CWSI=\frac{T_{c}-T_{wet}}{T_{dry}-T_{wet}}$$

T_c_ represented the actual temperature of the canopy, T_wet_ and T_dry_ were the lower and upper limits of canopy temperature. Thermal data were extracted using the ThermaCAM Researcher Pro 2.10 software (FLIR systems, Wilsonville, Oregon, USA) by manually selecting the regions of interest (ROIs), avoiding the empty spaces, and the wet and dry references. All temperature values corresponded to the average temperature of pixels within the selected area. In addition, according to Jones et al. (2002) a further index related to stomatal conductance (stomatal conductance index - Ig) was calculated by using the same references of CWSI, i.e., with the equation:


$$Ig=\frac{T_{dry}-T_{c}}{T_{c}-T_{wet}}$$

T_c_, T_dry_ and T_wet_ were the same parameters utilized in CWSI equation. Ig may also represent a reliable index for assessing plant water status, as it is theoretically proportional to g_s_, as observed in several studies (Jones et al., 2002; Costa et al., 2012; Belfiore et al., 2019). Thermal images were taken on 1, 8, 16, 22 and 29 August; 6, 11, 19 and 26 September; and 3 and 10 October.

#### 2.2.2 Fruit-based sensing

Fruit diameter was continuously monitored during the entire period, at 15‐min intervals, with the fruit gauges described by Morandi et al. (2007), wired to a CR‐1000 data logger (Campbell Scientific, Inc., Logan, UT, USA). Drupes were continuously measured using 10 fruit gauges, with one sensor per plant for a total of five plants per treatment. Fruit gauges were placed on sun exposed fruits at about 1.5 m from the ground (corresponding to medium canopy height). At the end of the measurement period, the fruit equatorial diameter was converted to fruit weight, as suggested by (Morandi et al., 2007). The following equation was used for the conversion:


$$W(g)=a\times D{\left(mm\right)}^{b}$$

where W was the fruit weight and D the diameter. For our fruit in the experiment, a and b were 0.003 (± 0.0004 S.E.) and 2.59 (± 0.052 S.E.), respectively, with R^2^ = 0.960. This equation was obtained by regressing diameter and weight data of 200 fruits randomly sampled from plants exposed to the two irrigation levels throughout the trial period. The stages of fruit growth were identified by monitoring the diameter and weight (Rallo and Rapoport, 2001; Trentacoste et al., 2010). Subsequently, fruit daily weight fluctuations (ΔW, g) and relative growth rate (RGR, mg g^-1^ min^-1^), were calculated. ΔW was obtained from the subtraction between the maximum and the minimum daily weight averaged for all monitored fruits. RGR was calculated from the absolute growth rate (AGR, g min^-1^) of individual fruits. In details, AGR and RGR were calculated as follows:


$$AGR=\frac{W_{1}-W_{0}}{t_{1}-t_{0}}$$

and


$$RGR=\frac{AGR}{W_{0}}$$

In the equations, W_1_ and W_0_ were the fruit weights at time t_1_ and t_0_, respectively. RGR provides an indication of dry mass accumulation in the fruit, while ΔW is primarily related to fruit water exchanges through the xylem and transpiration (Carella et al., 2021). Such parameters were correlated with Ψ_stem_ and g_s_ in order to evaluate how the fruits responded to changes in plant water status, in terms of water exchanges and carbohydrates uptake.

### 2.3 Remote sensing measurements

#### 2.3.1 Flight scheduling and multispectral data acquisition

A DJI Phantom 4 Multispectral UAV (DJI, Shenzhen, China) equipped with a multispectral camera was used to obtain the reflectance in different narrow bands of the electromagnetic spectrum of each plant. Specifically, the camera has six 1/2.9″ CMOS sensors, i.e. one RGB sensor and five monochrome sensors with band centers in Blue (B, 450 ± 16nm), Green (G, 560 ± 16nm), Red (R, 650 ± 16nm), RedEdge (RE, 730 ± 16nm) and Near InfraRed (NIR, 840 ± 26nm). Two flights were carried out on 19 September and 10 October with automatic flight configuration using the way-points and RTK mode for correcting the GNSS signal. The flights were performed at about 13:00 at an altitude of 70 m, generating a Ground Surface Distance (GSD) of 3.6 cm. Before each flight, a calibration panel and 10 Ground Control Points (GCP) were positioned and georeferenced with the Stonex S7-G instrument according to Roma et al. (2023). The image acquisition was made in stop-and-go mode with 70% front and side overlap ratio, while the gimbal pitch was set at 90° (downwards).

#### 2.3.2 Image processing

**Figure 2:**
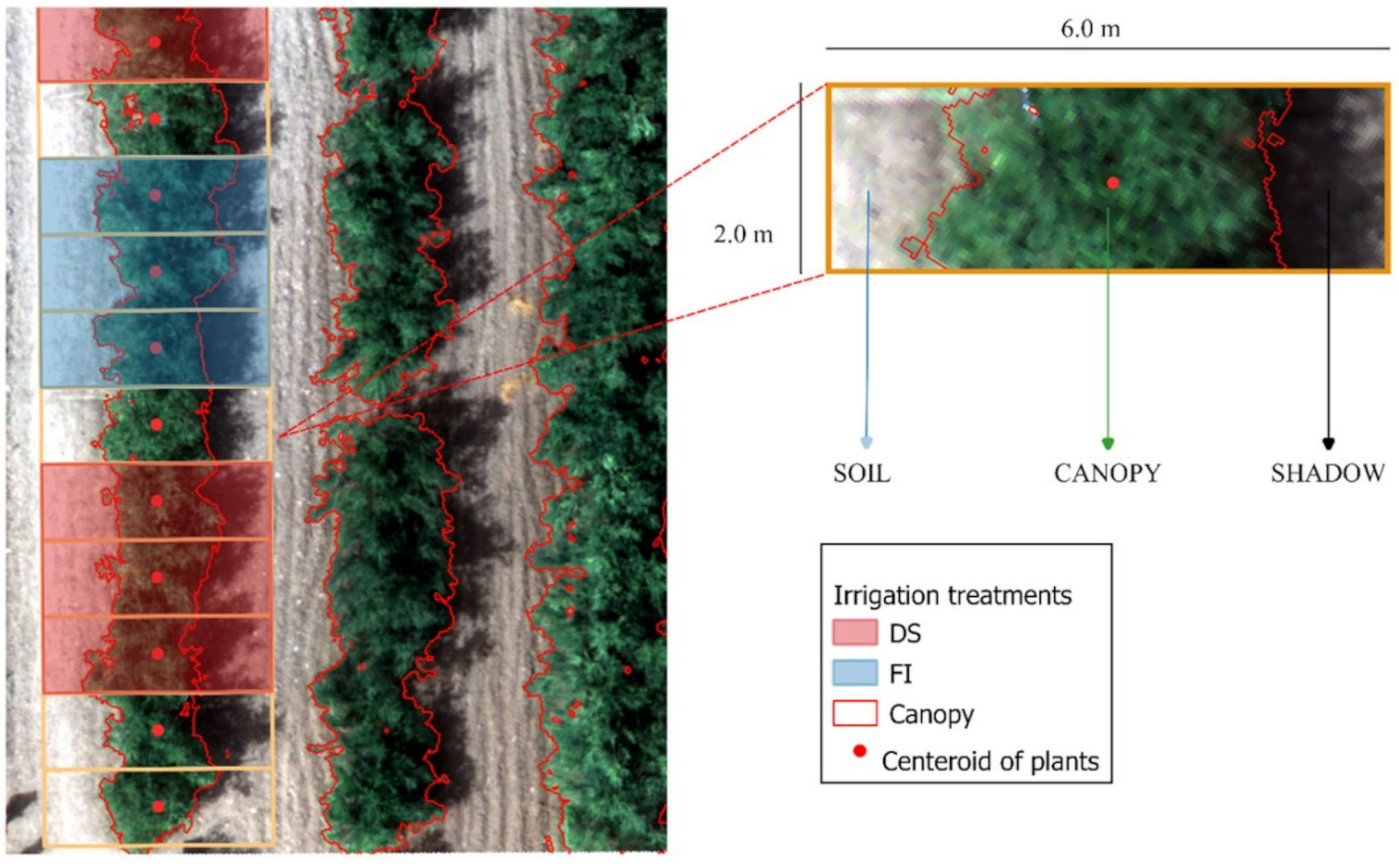
Representation of sub-plots used to obtain the spectral and geometric information per plant. FI, full irrigated; DS, drought stressed.

The photogrammetric reconstruction was carried out with Agisoft Photoscan Professional 1.7.3. (Agisoft Metashape, Saint Petersburg, Russia) using structure-from-motion (SfM) algorithms to obtain the multi-band orthomosaic and Digital Elevation Model (DEM). The OBIA (Object-Based Image Analysis) of geo-spatial and multispectral data was performed in the open-source software QGIS 3.2 (QGIS Geographic Information System). Specifically, orthomosaic segmentation and classification to separate the canopy from the background were carried out using the machine learning algorithm implemented in the Orfeo Tool Box (OTB tool). Once the canopies were obtained, the spectral information for each tree was extracted using Statistical Zone tools (Stateras and Kalivas, 2020; Roma et al., 2023). To obtain the information of each tree, a centroid of plants was identified, and a sub-plot of 6 x 2 m was built for each one (**Figure 2**). The digital numbers (DN) inside of each sub-plot were used to evaluate the spectral and geometric features per plant.

The spectral information was related to the determination of several water sensitive vegetation indices used in the literature (**Table 1**).

**Table 1:** Vegetation indices considered to determine tree water status, using wavelengths within the VIS-NIR range.

Vegetation index	Acronym	Equation	Reference
Chlorophyll Vegetation Index	*CVI*	(ρNIR/ρG) * (ρR+ρG)	Vincini et al. (2008)
Green Index	*GI*	ρG/ρR	Zarco-Tejada et al. (2005)
Green Normalized Difference Vegetation Index	*GNDVI*	(ρNIR−ρG)/(ρNIR+ρG)	Gitelson et al. (1996)
Modified Soil-Adjusted Vegetation Index	*MSAVI*	0.5 * {2 ρNIR +1 – SQRT[(2 ρNIR+1)^2^ – 8 (ρNIR – ρR)]}	Qi et al. (1994)
Normalized Differential Greenness Index	*NDGI*	(ρG−ρR)/(ρG+ρR)	Pôças et al. (2015)
Normalized Difference RedEdge Index	*NDRE*	(ρNIR−ρRedEdge)/(ρNIR+ρRedEdge)	Maccioni et al. (2001)
Normalized Difference Vegetation Index	*NDVI*	(ρNIR−ρR)/(ρNIR+ρR)	Rouse et al. (1974)
Water Index	*WI*	ρR/ρNIR	Peñuelas et al. (1993)

G, Green; R, Red; NIRm, Near Infrared spectral bands; ρ, reflectance.

### 2.4 Statistical analysis

The means of Ψ_stem_, g_s_ and P_n_ of FI and DS plants were compared by using repeated measure ANOVA at the 0.05 significance level using Jamovi 2.4.14 procedures (The Jamovi Project, 2023). Linear and nonlinear regression analysis were performed to relate the parameters obtained from proximal and remote sensing with Ψ_stem_ and g_s_ using Sigmaplot 14.0 (Systat Software Inc., Chicago, IL, USA) procedures. The remote sensing data were analyzed with two-way analysis of variance using date and irrigation levels as main factors, followed by Tukey’s *post hoc* test.

## 3 Results and discussion

### 3.1 Climate and irrigation data

**Figure 3:**
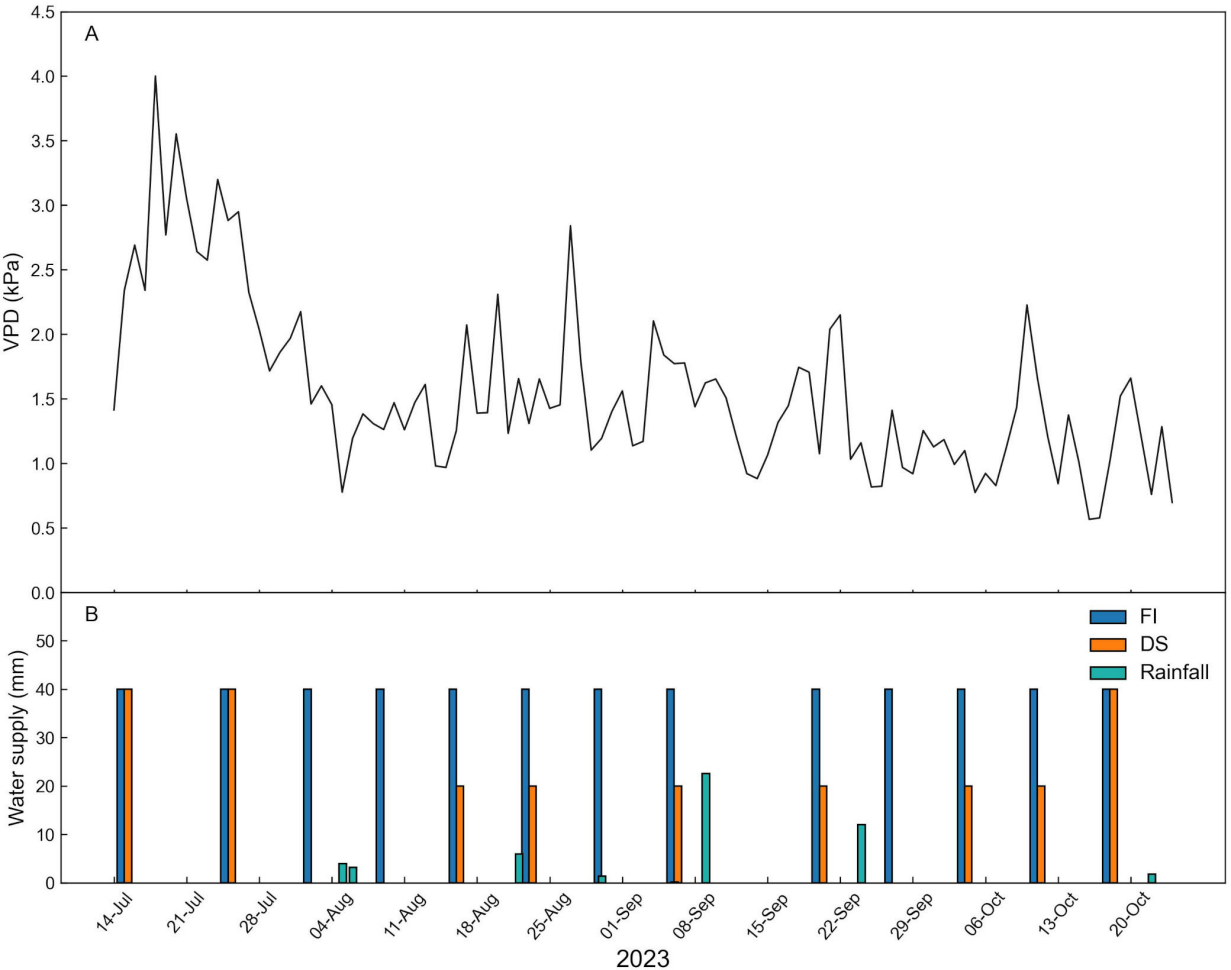
Daily mean vapor pressure deficit (VPD, **(A)** and water supply (irrigation and rainfall, **(B)** to full irrigated (FI), and drought stressed (DS) ‘Calatina’ olive trees.

As expected, the highest values of VPD were recorded in July. Specifically, the highest VPD was recorded on 18 July. In contrast, the lowest level was reached on 16 October (**Figure 3A**). Seven rainfall events occurred during the trial, totaling 51.2 mm of rain. The most intense rainfall events occurred on 8 and 23 September (22 mm and 12 mm, respectively). In FI trees, irrigation was carried out weekly with a total of 40 mm (**Figure 3B**), for a total of 571.2 mm (**Table 1**) (including rainfall). No irrigation was applied between 6 September and 19 September due to a district water shortage. In DS trees, emergency irrigation was carried out when Ψ_stem_ went below -4 MPa, and near fruit ripening, totaling 291.2 mm (including rainfall).

### 3.2 Tree water status and gas exchange

**Figure 4:**
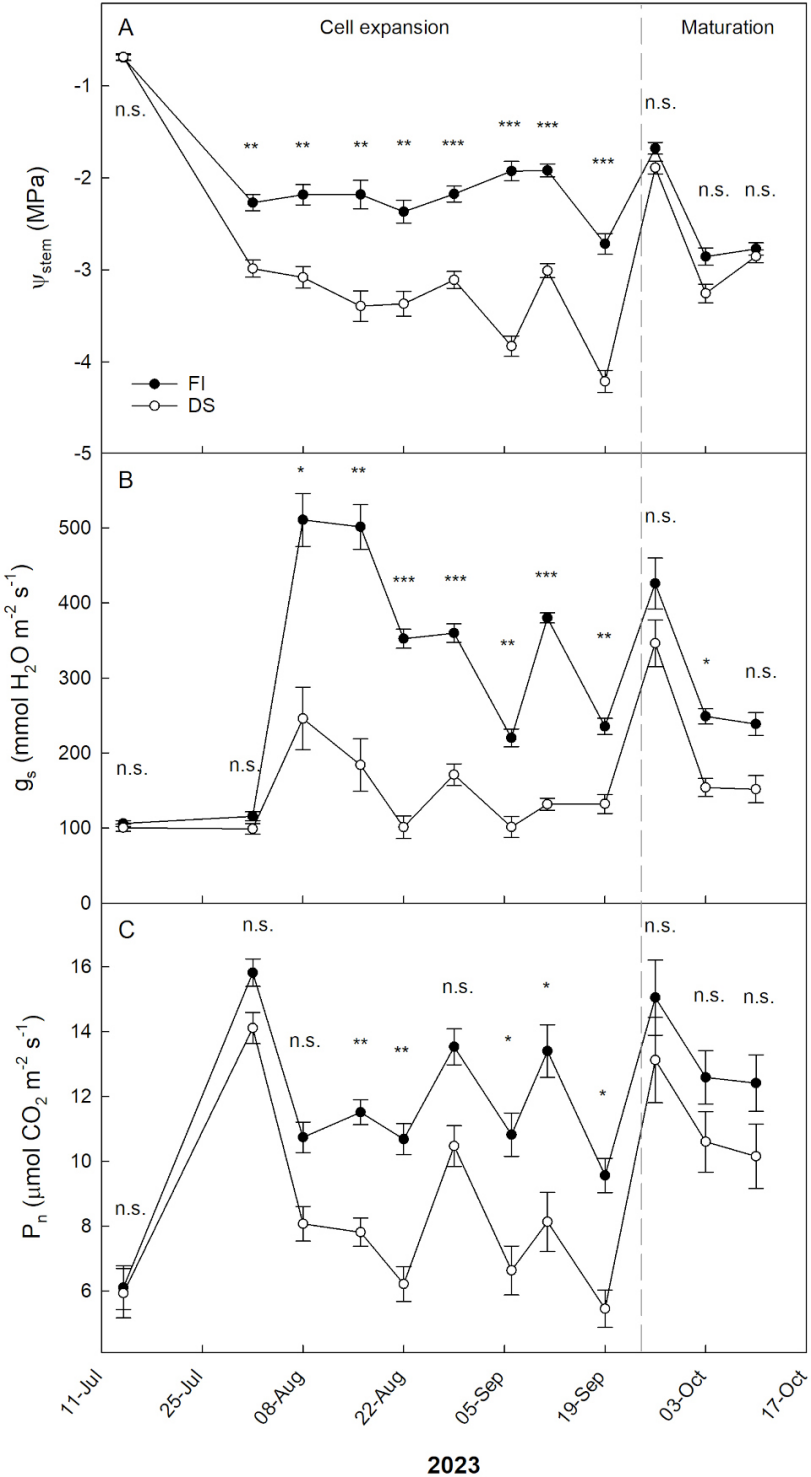
Trends of midday stem water potential (Ψ_stem_, **A**), stomatal conductance (g_s_, **B**) and net photosynthesis (P_n_, **C**) in ‘Calatina’ olive trees from 14 July to 10 October 2023. Black and white dots represent full irrigated (FI) and drought stressed (DS) trees, respectively. Error bars indicate standard errors of means. n.s., no significantly different; *, significantly different for P < 0.05; **, significantly different for P < 0.01; ***, significantly different for P < 0.001. The gray dashed line represents the threshold between cell expansion and maturation stage, corresponding to about 23 September 2023.

At the beginning of the trial, all trees received the same amount of irrigation water; indeed, no significant differences were found between FI and DS trees in terms of Ψ_stem_, g_s_, and P_n_ (**Figure 4**). Regarding Ψ_stem_, significant differences were observed between irrigation treatments from 1 August until 19 September (**Figure 4A**). No significant differences were observed from 26 September until the end of the experiment (10 October). On 26 September, Ψ_stem_ values were above -2 MPa in both FI and DS trees, due to both rewatering and a rainy event on 23 September. A slight increase in Ψ_stem_ in DS plants was observed on 29 August, following a light rainfall event. The lowest Ψ_stem_ level was recorded on 19 September in DS plants; however, a low value (< 2.5 MPa) was also observed in FI plants on the same date. This was due to a temporary water network failure, so no irrigation was carried out on 18 September. For this reason, irrigation was postponed to 20 September.

The trend of g_s_ over time was consistent with that of Ψ_stem_ for almost the entire experiment (**Figure 4B**). On 1 August, despite the differences in Ψ_stem_, no significant differences of g_s_ were observed between the two irrigation treatments. Most likely, hydration levels were not sufficiently low for the trees to exhibit different stomatal opening behavior. In subsequent dates, significant differences in g_s_ between irrigation treatments were observed until 19 September. After this date, despite the rainfall events and rewatering, ‘Calatina’ trees seemed to keep memory of the watering differences. Indeed, DS significantly reduced g_s_ on 3 October. This behavior may have been mediated by chemical signals like abscisic acid, possibly accumulated in the roots and transferred to the shoots where it causes a g_s_ reduction (Brito et al., 2020).

Regarding P_n_, no significant differences between the two irrigation treatments were observed until 8 August (**Figure 4C**). On this date, despite differences in g_s_, P_n_ of FI and DS trees was not statistically different. In other words, despite the reduction in stomatal closure, similar levels of CO_2_ were assimilated, suggesting an increase water use efficiency. This usually happens when stomatal closure is only partial and it decreases water loss more than CO_2_ intake due to the different partial pressures of the two gasses (Lawson and Blatt, 2014). On 15 and 22 August, P_n_ was significantly reduced by DS. As in Ψ_stem_, no significant differences of P_n_ were observed from 26 September until the end of the experiment.

**Figure 5:**
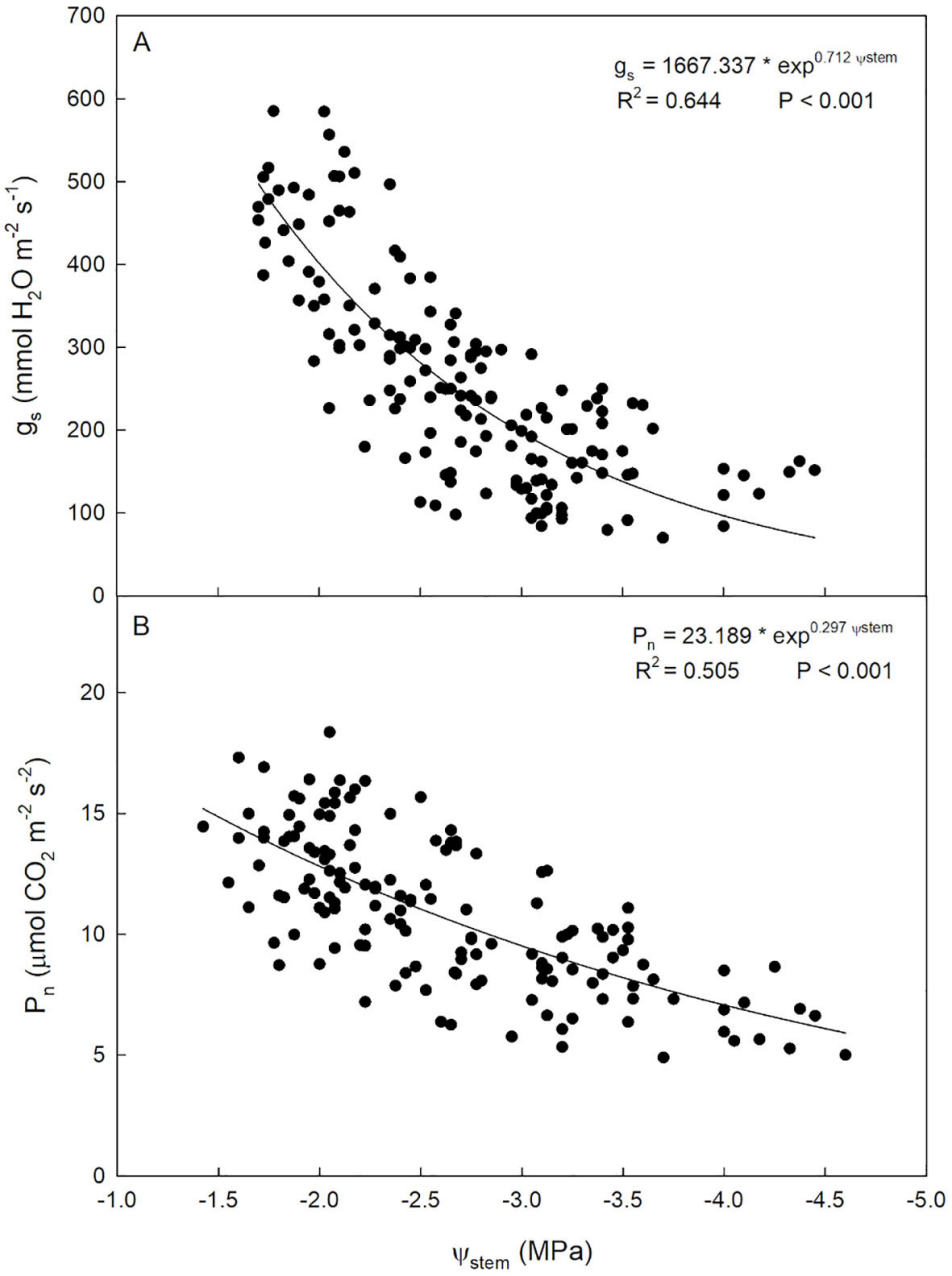
Relationships between stem water potential (Ψ_stem_) and stomatal conductance [g_s_, **(A)**] and net photosynthesis [P_n_, **(B)**] in ‘Calatina’ olive trees from 1 August to 10 October 2023.

Within the observed Ψ_stem_ range (from -4.7 to -1.6), the relationship with g_s_ was described by a direct exponential model (**Figure 5A**). Specifically, the model shows a strong direct relationship from -1.6 to -2.7 MPa, exhibiting a continuous decrease in g_s_ sensitivity as Ψ_stem_ decreases. In other words, as water stress progresses, stomatal response loses sensitivity and the stomatal closure is gradually lost; as a result, minimal transpiration is maintained. This behavior has been already reported in olive (Moriana et al., 2012; Marino et al., 2018).

P_n_ ranged from 4.9 to 18.3 µmol CO_2_ m^-2^ s^-1^. In the observed Ψ_stem_ range, the relationship between Ψ_stem_ and P_n_ was also best represented by a direct exponential model. As Ψ_stem_ decreased, P_n_ decreased more slowly, gradually reducing sensitivity to Ψ_stem_ (**Figure 5B**). This kind of relationship was also observed in ‘Arbequina’ olive (Marino et al., 2018; Ahumada-Orellana et al., 2019).

### 3.3 Proximal sensing

#### 3.3.1 Thermal imaging

**Figure 6:**
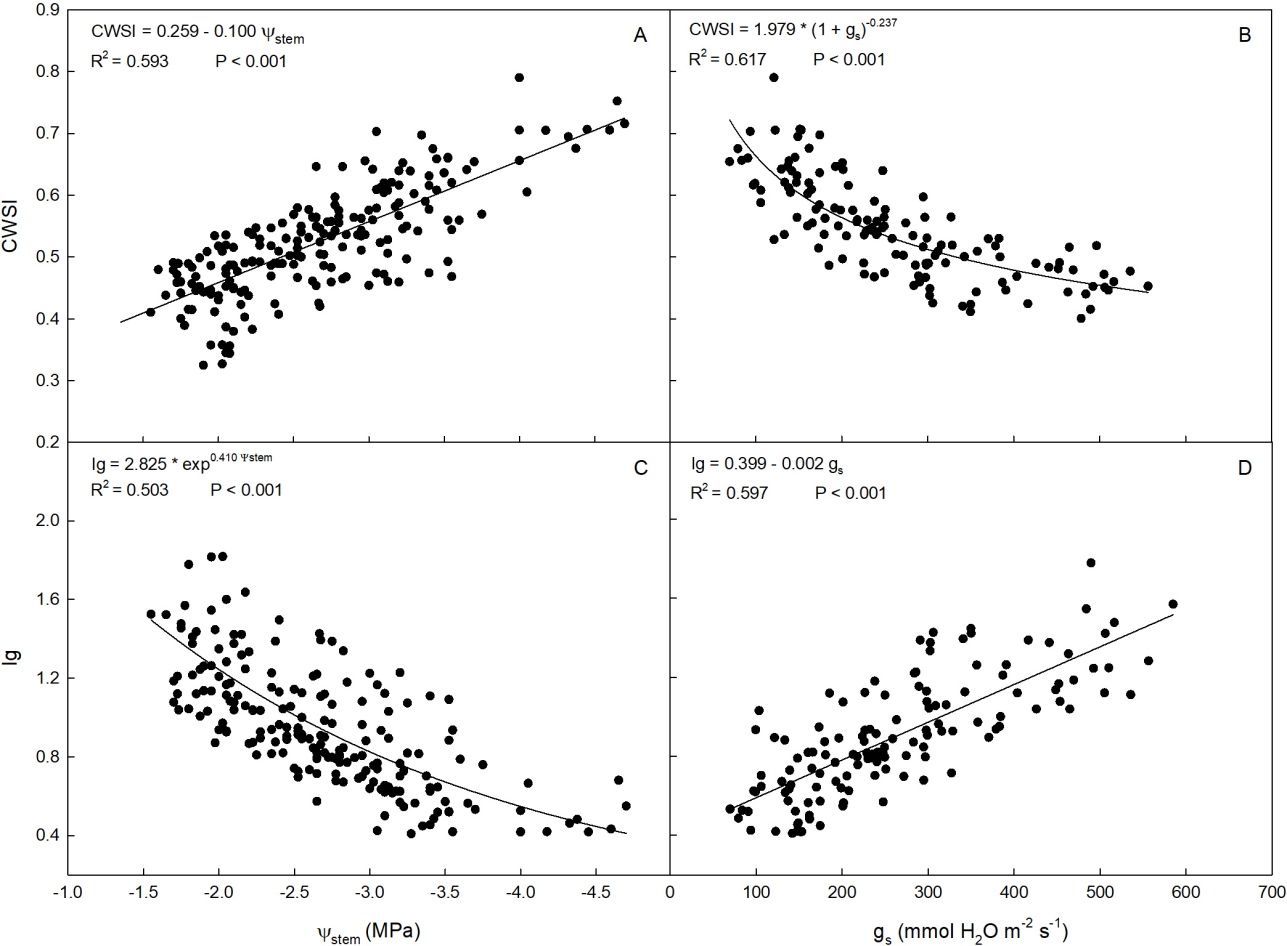
Relationships between stem water potential (Ψ_stem_) and crop water stress index [CWSI, **(A)**], stomatal conductance (g_s_) and CWSI **(B)**, Ψ_stem_ and stomatal conductance index [Ig, **(C)**], and g_s_ and Ig **(D)** in ‘Calatina’ olive trees from 1 August to 10 October 2023.

A negative linear relationship between CWSI and Ψ_stem_ was observed (**Figure 6A**). CWSI ranged from approximately 0.35 to 0.8 MPa, while Ψ_stem_ ranged from about -1.6 to -4.7 MPa. This indicates that canopy temperature normalized with CWSI is proportional to changes in the water status of ‘Calatina’ trees. Therefore, CWSI calculated using thermal imagery proved to be a useful parameter for plant water stress assessment, serving as an alternative to the more laborious measurement of Ψ_stem_ by using the pressure chamber. Similar ranges and relationships between Ψ_stem_ and CWSI have been documented in ‘Arbequina’ (Egea et al., 2016) and in ‘Barnea’ and ‘Cobrançosa’ (Ben-Gal et al., 2009; Marques et al., 2023) olive. Caruso et al. (2022b) found a significant relationship between remotely sensed CWSI and Ψ_stem_ in ‘Frantoio’ and ‘Leccino’ olive, with values comparable to those observed in this study. Linear relationships between the two parameters have also been documented in other fruit species such as cherry (Blaya-Ros et al., 2020), apple (Mohamed et al., 2021), peach (Ramírez-Cuesta et al., 2022) and grapevine (Pou et al., 2014).

On the other hand, the relationship between CWSI and g_s_ followed an inverse exponential model (**Figure 6B**) similar to the one between Ψ_stem_ and g_s_. In this case, CWSI increased almost linearly as g_s_ decreased up to approximately 200 mmol H_2_O m^-2^ s^-1^. Below this value, CWSI was more sensitive to g_s_ changes, and increased more rapidly. To date, mainly linear relationships between CWSI and g_s_ have been documented in olive (García-Tejero et al., 2017; Marques et al., 2023). However, due to the small number of olive cultivars on which this relationship has been studied, it is likely that the latter may vary depending on the cultivar.

The relationship between g_s_ and Ψ_stem_ also followed a direct exponential function (**Figure 5A**). Since Ig was developed as thermal index for estimation of stomatal conductance, the relationship between Ig and Ψ_stem_ (**Figure 6C**) was similar to the one between Ψ_stem_ and g_s_. Specifically, from -1.6 toward -4.7 MPa there was a tendency of Ig to lose sensitivity to changes in Ψ_stem_ as the latter decreased.

A positive linear relationship between Ig and g_s_ was observed (**Figure 6D**), confirming that Ig is a more direct estimator of stomatal conductance. Similar relationships were found in several studies with other species (Jones et al., 2002; Reinert et al., 2012; Yu et al., 2015).

#### 3.3.2 Fruit based-sensing

**Figure 7:**
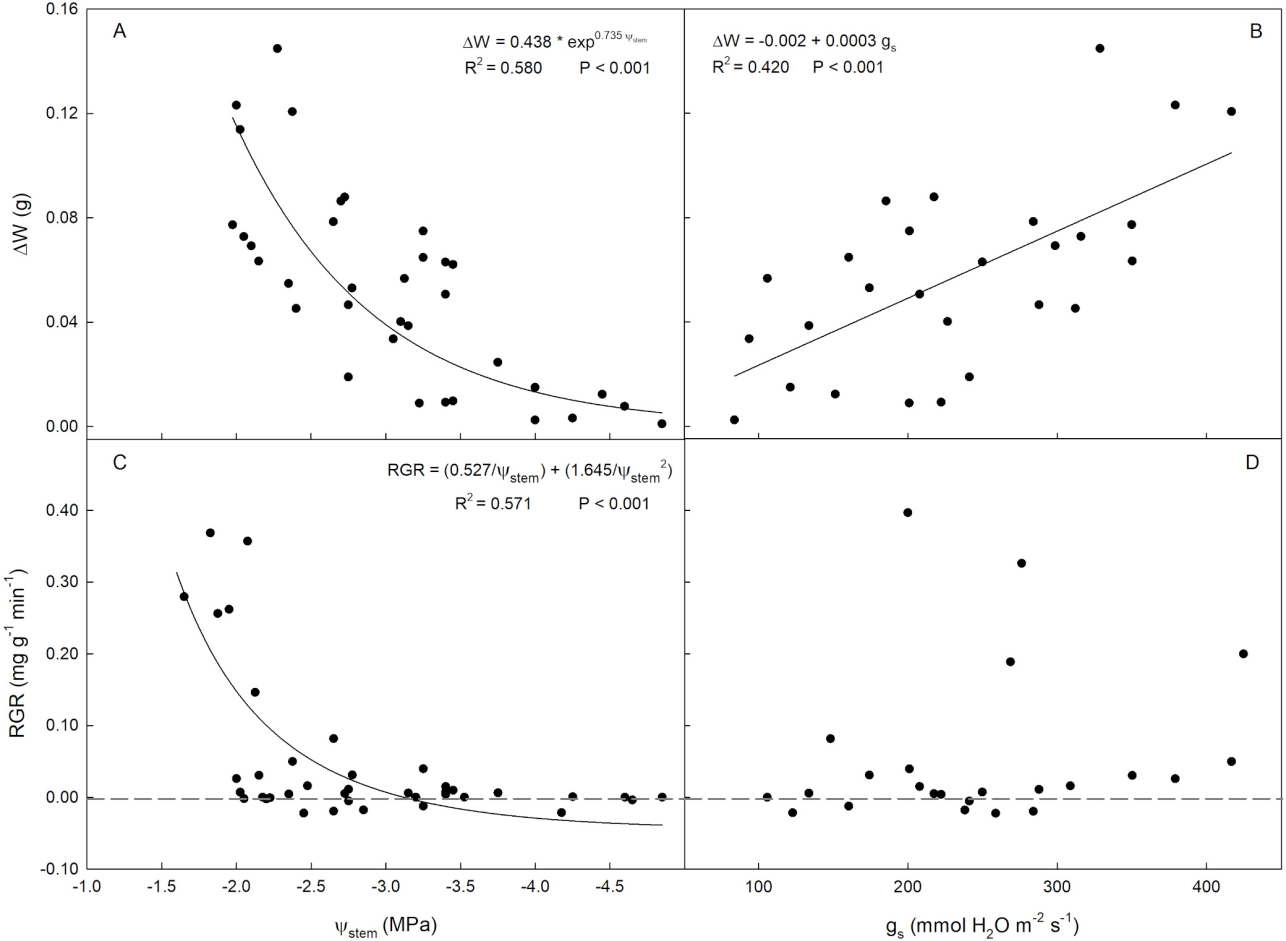
Relationships between fruit daily weight fluctuations (ΔW) and stem water potential [Ψ_stem_, **(A)**], ΔW and stomatal conductance [g_s_, **(B)**], fruit relative growth rate [RGR, **(C)**] and Ψ_stem_, and RGR and g_s_ **(D)** in ‘Calatina’ olive trees from 1 August to 26 September 2023.

For associations with ΔW and RGR only the period of cell expansion was taken into account, as the fruit exhibits minimal response to changes in water status during the maturation stage. In fact, during maturation stage, internal and external changes in the fruit texture, flavor, and color prevail over water exchanges (Giovannoni, 2001; Carella et al., 2021). On the other hand, when the fruit is in the stage of cell division, phloem and xylem contribution are similar (Morandi et al., 2007). During this phase, carbohydrate intake is essential. They are mainly sourced from actively photosynthesizing leaves and transported into the fruit through the phloem (Génard et al., 2008). On the contrary, during pit hardening stage, fruit water flows are very limited. At this stage, water deficit do not affect fruit growth (Goldhamer, 1997; Moriana et al., 2003; Corell et al., 2022). At fruit cell expansion stage, a direct exponential relationship between ΔW and Ψ_stem_ was observed (**Figure 7A**). Specifically, as Ψ_stem_ increased, fruit weight fluctuations become more and more pronounced, increasing its response sensitivity. At Ψ_stem_ values below -2.5 MPa, ΔW began to lose response sensitivity, suggesting that below this threshold fruit water flows tended to stabilize. This relationship between ΔW and Ψ_stem_ suggests that below this Ψ_stem_ value, the drupe may promptly respond to water stress by enhancing its water retention capacity. Specifically, a gradual fruit stomatal closure may be occurred, decreasing fruit transpiration rate and maintaining appropriate tissue hydration (Lescourret et al., 2001; Morandi et al., 2010). Secondly, a fruit osmotic adjustment may be occurred, leading to the accumulation of solutes capable of decreasing the osmotic potential and contributing to maintain tissues hydration. In olive, fruit osmotic adjustment was documented by Girón et al. (2015) in cv. Manzanillo. To date, few studies have correlated fruit ΔW and Ψ_stem_. In ‘Arbequina’ olive, Fernandes et al. (2018) observed a linear relationship between the two parameters in full irrigated plants, while no relationship was observed in trees under water deficit. Furthermore, it is worth noting that the ‘Calatina’ fruit maintained a high water exchange capacity even when the Ψ_stem_ ranged from about -3 to -2.5 MPa (mild water stress condition).

A positive linear relationship was observed between ΔW and g_s_ (**Figure 7B**). However, such relationship proved to be less tight than the relationship between Ψ_stem_ and ΔW. A direct exponential relationship was also observed between RGR and Ψ_stem_ (**Figure 7C**). It is interesting to note that RGR is markedly higher at Ψ_stem_ levels between -2 and -1.6 MPa, further suggesting that maintaining plants within that range ensures optimal fruit growth rates. Conversely, RGR began to approach zero at Ψ_stem_ of about -3 MPa, losing sensitivity to further decreases of Ψ_stem_, and reaching slightly negative values at Ψ_stem_ below -3 MPa. In other studies, mainly linear relationships between fruit growth rate and Ψ_stem_ were reported (Boini et al., 2019; Scalisi et al., 2020; Marino et al., 2021). However, the range of Ψ_stem_ examined was narrower than in this study.

No significant relationship was found between RGR and g_s_, suggesting that fruit growth mainly responded to changes in Ψ_stem_ rather than g_s_ (**Figure 7D**). In ‘Gala’ apple, Boini et al. (2019) studied the correlations between fruit growth parameters, Ψ_stem_ and gas exchanges. The Authors found that the fruit growth rate exhibited the strongest correlation with Ψ_stem_. Interestingly, the fruit daily size fluctuations, in contrast to this study, showed a stronger relationship with g_s_ rather than with Ψ_stem_. This discrepancy may stem from measurements taken during the late stages of apple cell expansion, where factors influencing fruit external and internal changes may differ from olive. For example, in apples, simple sugar accumulation prevails, while in olives, oil accumulation and oleuropein degradation occur (Tombesi, 1993; Tijero et al., 2021). As expected, no significant relationships between fruit growth parameters and Ψ_stem_ or g_s_ were identified after the cell expansion stage in our study.

### 3.4 Remote sensing

**Figure 8:**
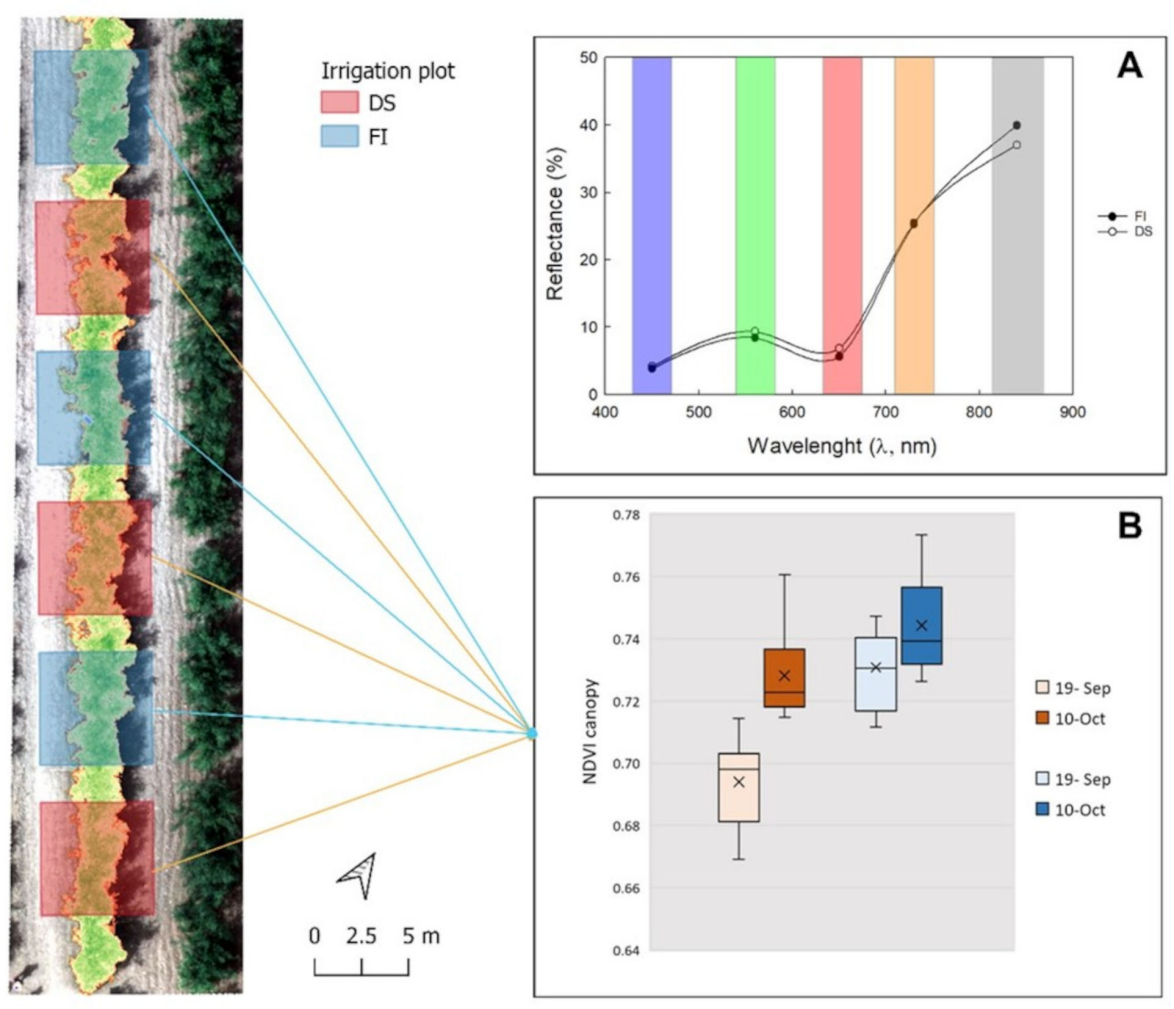
Representation of canopy NDVI along a tree row for full irrigation (FI) and drought stress (DS) treatments. The frequency histogram **(A)** concerns the first acquisition date (19 September) while the box-plot **(B)** shows the NDVI values on 19 September and 10 October 2023 for both treatments.

The experiments allowed the investigation of the spectral conditions of each plant as a function of Ψ_stem_. Specifically, different behavior was observed in the different bands and consequently also in the vegetation indices (**Figure 8**). In the VIS and NIR zones, the average reflectance of the vegetation showed the typical plant trend. Indeed, in the visible zone of the electromagnetic spectrum (400 – 700 nm), a higher reflectance of DS plants compared to FI plants was observed (**Figure 8A**). In the NIR zone (700 – 900 nm), the opposite trend was observed. Specifically, the reflectance in the bands of blue, green, red, RedEdge and NIR were 3.8% 8.4%, 5.6%, 25.3% and 38%, respectively in the FI trees, while they were 4.1%, 9.02%, 6.3%, 25.4% and 37% in the DS trees. Similar reflectance patterns have also been observed in other studies conducted on olive trees (Rallo et al., 2014) as well as other crops (Pôças et al., 2017). NDVI is considered the reference vegetation index for plant vigor and health. In this regard, the differences between FI and DS plants are evident only on September 19 (**Figure 8B**), where the Ψ_stem_ range was wider (**Figure 4A**).

All the indices were higher in FI than in DS plants, except for the WI (**Table 2**). Indeed, while all the other indices are used to estimate plant growth and health conditions (Noori and Panda, 2016; Jorge et al., 2019; Jurado et al., 2020; Stateras and Kalivas, 2020), the WI is an index directly related to water stress detection (Peñuelas et al., 1993; Pôças et al., 2017). Furthermore, an increase of all the indices is observed as the season progresses. All indices were able to differentiate between the two irrigation treatments at the time of greater water stress (19 September), lower Ψ_stem_. Furthermore, only NDRE, GNDVI, WI, NDGI, and GI were able to differentiate the two treatments also in October.

**Table 2:** Vegetation indices for ‘Calatina’ olive trees on 19 September and 10 October.

	NDVI ^a^	NDRE	GNDVI	CVI	MSAVI	WI	NDGI	GI
19 September								
DS	0.692 ± 0.012	0.191 ± 0.005	0.613 ± 0.014	2.891 ± 0.088	0.495 ± 0.031	0.171 ± 0.011	0.184 ± 0.014	1.441 ± 0.022
FI	0.733 ± 0.014	0.201 ± 0.006	0.639 ± 0.014	3.038 ± 0.121	0.521 ± 0.022	0.149 ± 0.009	0.202 ± 0.011	1.488 ± 0.029
	*	**	**	*	*	**	*	**
10 October								
DS	0.733 ± 0.022	0.210 ± 0.007	0.639 ± 0.013	3.051 ± 0.140	0.505 ± 0.040	0.147 ± 0.009	0.190 ± 0.023	1.481 ± 0.038
FI	0.752 ± 0.019	0.233 ± 0.012	0.660 ± 0.020	3.131 ± 0.121	0.553 ± 0.038	0.129 ± 0.022	0.220 ± 0.034	1.564 ± 0.073
	n.s.	**	*	n.s.	n.s.	*	*	*

When present, letters denote significant differences between full irrigated (FI) and drought stressed (DS) trees (P < 0.05). n.s., no significantly different; *, significantly different for P < 0.05; **, significantly different for P < 0.01. NDVI, normalized difference vegetation index; NDRE, normalized difference RedEdge index; GNDVI, green normalized difference vegetation index; CVI, chlorophyll vegetation index; MSAVI, modified soil-adjusted vegetation index; WI, water index; NDGI, normalized difference greenness index; GI, green index.

The linear relationships between indices calculated from UAV multispectral camera and Ψ_stem_ on 19 September and 10 October were split by date because they showed significantly different slopes as a function of date (**Table 3**). In detail, significant differences between slopes were found in all the vegetation indices taken into account. This can be explained because on 10 October, trees were more hydrated, which reduced the measured range of Ψ_stem_, making the changes in indices not easily appreciable. Indeed, on 10 October, the regressions slopes were less steep than those on 19 September.

**Table 3:** Comparison of the regression slopes for the relationships between vegetation indices and stem water potential (Ψ_stem_) on 19 September and 10 October in ‘Calatina’ olive trees (t-test, P < 0.05).

	Slope		P-value
	19 September	10 October	
NDVI	0.018	0.106	0.008
MSAVI	0.016	0.126	0.004
NDRE	0.008	0.047	0.014
NDGI	0.01	0.107	0.005
GNDVI	0.017	0.088	0.007
WI	-0.012	-0.072	0.003
CVI	0.106	0.397	0.033
GI	0.031	0.348	0.005

**Figure 9:**
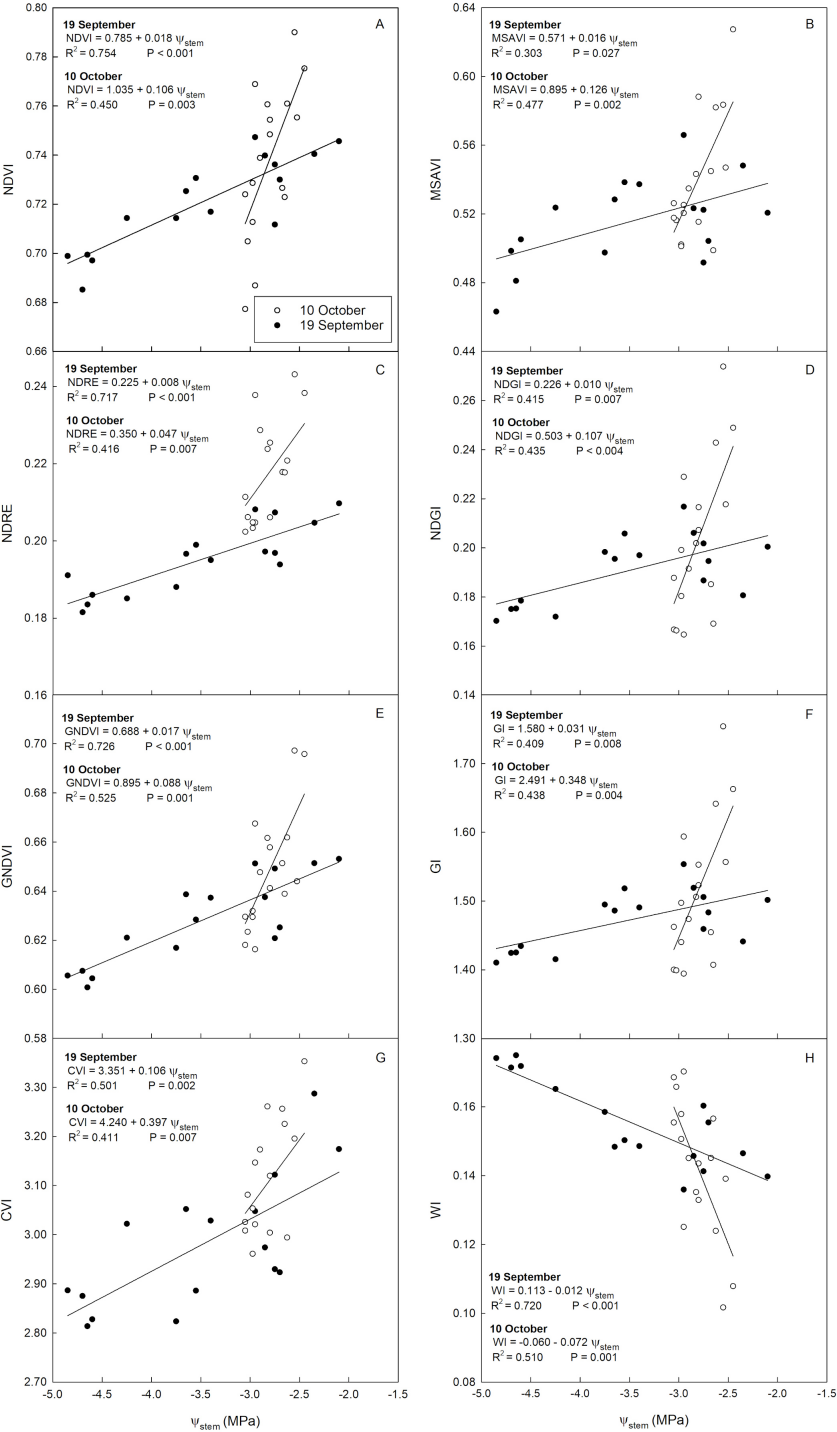
Linear relationships between stem water potential (Ψ_stem_) and normalized difference vegetation index [NDVI, **(A)**], modified soil-adjusted vegetation index [MSAVI, **(B)**], normalized difference RedEdge index [NDRE, **(C)**], normalized difference greenness index [NDGI, **(D)**], green normalized difference vegetation index [GNDVI, **(E)**], green index [GI, **(F)**], chlorophyll vegetation index [CVI, **(G)**], water index [WI, **(H)**] on 19 September (black dots) and 10 October (white dots) 2023.

A strong and significant positive linear relationship was found between NDVI and Ψ_stem_ on 19 September (**Figure 9A**), while a weaker but significant linear relationship was observed on 10 October. NDVI is a vegetation index closely dependent on chlorophyll content and leaf cell structures. Specifically, chlorophyll has a strong absorption peak in the red region, while leaf mesophyll constituents and canopy structure are the factors that positively influence canopy NIR reflectance (Gitelson, 2004; Caruso et al., 2023). Indeed, increases in NIR reflectance correlate with increases in leaf thickness (Castro and Sanchez-Azofeifa, 2008). Thus NDVI is mainly used for the assessment of changes in canopy biophysical properties (leaf area index, fraction of absorbed photosynthetically active radiation, chlorophyll content, etc.) (Gitelson, 2004). These characteristics are closely related to the water status of the plant, as water is the main component of metabolic processes (e.g., photosynthesis) that determine chlorophyll content and canopy structure (Hailemichael et al., 2016). Since these parameters are closely related to plant water status, NDVI serves as a reliable indirect indicator of plant water status. Similar relationships in olive were found by Marino et al. (2014) and Rallo et al. (2014).

Also MSAVI was linearly related to Ψ_stem_ on both dates, but reported the lowest R^2^ on 19 September and one of the highest R^2^ on 10 October (**Figure 9B**). To date, mixed results have been found in the literature on the effectiveness of the MSAVI and similar soil-adjusted indexes for assessing plant water status. In grapevine, Romero et al. (2018) reported a stronger correlation between MSAVI and Ψ_stem_ compared to NDVI. Conversely, Conesa et al. (2019) observed a weaker relationship in nectarine. Since this is an index that tends to remove the soil effect, the effectiveness of removing this effect may depend on different soil characteristics, as the spectral signature of the soil background varies with color, moisture, texture, etc (Baumgardner et al., 1986). Ren and Feng (2015) compared MSAVI with two soil unadjusted vegetation indices (NDVI and simple ratio index, SR), finding that the performance of MSAVI for estimating above-ground vegetation is lower than NDVI and SR.

Relationships of Ψ_stem_ with NDRE were similar to those with NDVI (**Figure 9C**). Since NDRE is calculated using the RedEdge band, it turns out to be a sensitive indicator of changes in chlorophyll content, thus indirectly related to plant water status (Boiarskii and Hasegawa, 2019; Roma et al., 2023). Similar relationships have been observed in the literature in other tree species such as sweet cherry (Blanco et al., 2020) and grapevine (Tang et al., 2022).

Relationships with NDGI showed relatively low R^2^ values on both dates (**Figure 9D**). This result partly disagrees with results found in other studies, which instead emphasize its reliability (Rallo et al., 2014; Pôças et al., 2015). However, since it is a greenness index, it may be influenced by factors like canopy exposure, e.g., paraheliotropism, as suggested by Rallo et al. (2014). In addition, the NIR band, which is highly sensitive to changes in the plant water status, is not used to calculate NDGI (Raz et al., 2003).

A strong significant linear relationship was found between GNDVI and Ψ_stem_ on both 19 September and 10 October (**Figure 9E**), suggesting that this vegetation index may be one of the most accurate in estimating ‘Calatina’ tree water status. GNDVI, taking into account the Green band, is positively correlated with anthocyanin content and negatively correlated with chlorophyll content, making it very sensitive to abiotic stresses (Viña and Gitelson, 2010). Since GNDVI is very sensitive to variations in chlorophyll content, the stronger correlation observed on 10 October compared to other indices can be attributed to the pronounced impact of light changes on chlorophyll content. Indeed, photosynthetically active radiation (PAR) on 10 October was 1633.55 ± 195.11 µmol m^-2^ s^-1^ (in contrast to 1121.15 ± 205.50 µmol m^-2^ s^-1^ recorded on 19 September). To date, there are not many studies on the assessment of olive tree water status using GNDVI. Rallo et al. (2014) found a significant relationship between GNDVI and Ψ_stem_ in ‘Nocellara del Belice’ olive, however, the coefficient of determination (R^2^ = 0.41) was lower than in this study. Contrarily, numerous studies on grapevines have consistently demonstrated that GNDVI is one of the most reliable indirect predictors of Ψ_stem_ (Helman et al., 2018; Cogato et al., 2022; Caruso and Palai, 2023).

Finally, CVI and GI were indices that correlated weakly with Ψ_stem,_ both on 19 September and 10 October (**Figures 9G, H**). Similarly to NDGI, they are closely related to greenness, so they can be easily affected by canopy exposure (Vincini et al., 2008; Rallo et al., 2014). Although CVI is a good indicator of chlorophyll status, it has been little used for plant water status assessment. Andrade Junior et al. (2022) found a significant linear relationship between CVI and Ψ_stem_ in soybean, but weaker than NDVI and optimized soil adjusted VI (OSAVI). GI, on the other hand, has been used in several experiments. In olive, Rallo et al. observed a relationship with similar coefficient of determination (R^2^ = 0.45). In ‘Cabernet Sauvignon’ grapevine, the relationship between GI and Ψ_stem_ was weak (Romero et al., 2018), while in Grenache and Shiraz grapevines, it proved to be a good indicator of drought stress (Cogato et al., 2022).

WI was one of the indices that showed a strong, but negative relationship with Ψ_stem_ on both dates (**Figure 9F**). This occurred since it is given by the ratio of the Red to NIR reflectance. Thus, as water stress increases, Red reflectance increased and NIR reflectance decreased. Since this is an index closely dependent on NIR reflectance, it is often correlated with leaf relative water content (RWC) (Peñuelas et al., 1994). The reflectance in NIR is associated with plant cell structures, particularly the cell wall. Consequently, alterations in plant water status involving changes in cell turgor affect cell wall structure and ultimately NIR reflectance (Peñuelas et al., 1993). However, it is important to note that under prolonged water stress conditions, plants may adjust osmotically, which can affect the correlation between WI and Ψ_stem_. Similar results in olive were found by Asgari et al. (2023) and Marino et al. (2014). On the other hand, Rallo et al. (2014) observed a weaker relationship between WI and Ψ_stem_. WI was also found to be a good predictor of Ψ_stem_ in grapevine (Serrano et al., 2010) and ‘Satsuma’ mandarin (Dzikiti et al., 2010).

In summary, from highest to lowest R^2^, NDVI, GNDVI, WI, and NDRE were the indices that correlated best with Ψ_stem_, especially when the range of hydration considered was wide. In contrast, CVI, NDGI, GI and MSAVI were worse predictors of Ψ_stem_ than the aforementioned (for a more detailed report on the regressions, see **Supplementary Table 1**). Considering the higher reliability of Ψ_stem_ as the primary parameter for assessing plant water status, correlations between all the vegetation indices and g_s_ were not reported.

### 3.5 Combination of proximal and remote sensing

Combination of proximal and remote sensing data was explored by correlation analysis only on September 19 (the date when a wide range of Ψ_stem_ was covered) (**Table 4**).

**Table 4:** Pearson’s correlation analysis between proximal and remote sensing indices.

		NDVI	NDRE	GNDVI	WI	CVI	MSAVI	NDGI	GI
**CWSI**	r	-0.827	-0.730	-0.761	0.813	-0.559	-0.678	-0.698	-0.694
	P-value	0.000	0.005	0.003	0.001	0.047	0.011	0.008	0.009
**Ig**	r	0.743	0.624	0.682	-0.680	0.596	0.533	0.505	0.502
	P-value	0.006	0.030	0.015	0.015	0.041	n.s.	n.s.	n.s.
**ΔW**	r	0.593	0.614	0.670	-0.716	0.508	0.457	0.692	0.690
	P-value	n.s.	n.s.	n.s.	0.041	n.s.	n.s.	0.050	n.s.

CWSI, crop water stress index; Ig, stomatal conductance index; ΔW, fruit daily weight fluctuation; NDVI, normalized difference vegetation index; NDRE, normalized difference RedEdge index; GNDVI, green normalized difference vegetation index; CVI, chlorophyll vegetation index; MSAVI, modified soil-adjusted vegetation index; WI, water index; NDGI, normalized difference greenness index; GI. green index.

Correlations are significant for P < 0.05; n.s., non-significant.

Regarding thermal indices, the tightest correlations were observed between CWSI and NDVI and between CWSI and WI, indicating that NDVI and WI are the best vegetation indices for combining proximal CWSI and multispectral remote sensing indices. A strong inverse correlation was also found between CWSI and NDRE and between CWSI and GNDVI, suggesting that these indices may be a valid alternative to NDVI and WI for assessing ‘Calatina’ olive water status. This confirms the results reported in section 3.4.

Significant correlations were also observed between Ig and NDVI, GNDVI, WI, NDRE, and CVI (**Table 4**). In contrast, correlations between Ig and MSAVI, NDGI, and GI were not significant. Overall, these findings indicate that CWSI is a reliable indicator of ‘Calatina’ olive water status when combined with various multispectral vegetation indices.

As for ΔW, the only significant close correlations were found with WI and NDGI (**Table 4**), showing that WI is a recurrent and solid index for estimating the water status of ‘Calatina’ olive.

In summary, results suggested two possible combinations. The first involved the use of CWSI and NDVI or WI. This represents a more simplified solution, as it uses only two parameters, but is probably less accurate since these indices are related only to canopies. The other possibility involved CWSI and ΔW as proximal indices, and WI as a multispectral index. Indeed, WI correlates best with both CWSI and ΔW.

Although this approach uses multiple parameters, it would provide more comprehensive information about tree water status. Specifically, this combination integrates spatial and temporal scale information related to both canopy and fruit. Since these results were obtained under limited conditions (one cultivar, on a specific date, and in a specific geographic area), further studies are needed to validate this combined system.

## 4 Conclusions

The following trial aimed to combine proximal and remote sensing techniques to show the benefits of using both monitoring methods, while simultaneously assessing the water status and response of ‘Calatina’ olive under two distinct irrigation regimes.

Indices obtained from thermal imaging, continuous fruit monitoring and remote sensing showed significant relationships in most cases. In detail, CWSI calculated using the direct method proved to be a reliable indicator of both ψ_stem_ and g_s_. While Ig correlated better with g_s_, thus proving to be a good indicator of stomatal conductance. As for the ΔW and RGR of the fruit, calculated by continuous monitoring of fruit gauges, both parameters increase exponentially as ψ_stem_ increases. In particular, ΔW was found to be the index that correlates most strongly with ψ_stem_ during the trial period. These data can provide valuable insights into the temporal dynamics of fruit growth, helping to identify the onset of water stress in the drupe. This could represent a probable time when to irrigate. Regarding vegetation indices obtained from multispectral data, NDVI, GNDVI, WI and NDRE were found to be the vegetation indices that correlate best with ψ_stem_, achieving high levels of accuracy. Useful information on the spatial variability of the olive orchard related to plant water status can be obtained with these vegetation indices. This information can subsequently serve both for strategic placement of proximal sensors and for managing irrigation differently within the orchard considering the spatial variability. Finally, the combination of proximal and remote sensing data identified either the combined use of CWSI and NDVI or WI as a more simplified possibility, or the combination of CWSI, ΔW, and WI as a more comprehensive and informative system. Further studies on combining proximal and remote sensing data will be necessary to validate the system and establish intervention thresholds. This should also include monitoring other plant organs (leaf, trunk) and providing quantitative indication on irrigation volume. Incorporating sensors to measure sap flow or leaf transpiration could offer valuable information about plant water usage.

## Data Availability

The raw data supporting the conclusions of this article will be made available by the authors, without undue reservation.
