# Supplementary material for: Combining proximal and remote sensing to assess ‘Calatina’ olive water status
Source: Front Plant Sci. 2024 Aug 20;15:1448656. doi: 10.3389/fpls.2024.1448656 (PMC11368777; doi:10.3389/fpls.2024.1448656)
Supplement: Supplementary file 1 [file Table1.docx]

Supplementary Material

# Supplementary Figures and Tables

**Table S1.** Linear regression between stem water potential (Ψ_stem_) and normalized difference vegetation index (NDVI), modified soil-adjusted vegetation index (MSAVI), normalized difference RedEdge index (NDRE), normalized difference greenness index (NDGI), green normalized difference vegetation index (GNDVI), green index (GI), chlorophyll vegetation index (CVI), water index (WI) on 19 September and 10 October 2023.

|  | **19 September** | | | **10 October** | | |
| --- | --- | --- | --- | --- | --- | --- |
|  | **RMSE** | **R^2^** | ***P*-value** | **RMSE** | **R^2^** | ***P*-value** |
| **NDVI** | 0.010 | 0.754 | < 0.001 | 0.024 | 0.450 | 0.003 |
| **MSAVI** | 0.023 | 0.303 | 0.027 | 0.027 | 0.477 | 0.002 |
| **NDRE** | 0.005 | 0.717 | <0.001 | 0.011 | 0.416 | 0.007 |
| **NDGI** | 0.011 | 0.415 | 0.007 | 0.025 | 0.435 | 0.004 |
| **GNDVI** | 0.010 | 0.726 | <0.001 | 0.017 | 0.525 | 0.001 |
| **GI** | 0.034 | 0.409 | 0.008 | 0.080 | 0.438 | 0.004 |
| **CVI** | 0.099 | 0.501 | 0.002 | 0.093 | 0.411 | 0.007 |
| **WI** | 0.007 | 0.720 | <0.001 | 0.014 | 0.510 | 0.001 |

Relationships are significant for P < 0.05.
